# Suggested guidelines in reporting results from mediation analysis, standardized or unstandardized?

**DOI:** 10.1371/journal.pone.0310429

**Published:** 2024-09-18

**Authors:** Mohammad Nahian Ferdous Abrar, Hongmei Zhang, Yu Jiang

**Affiliations:** Division of Epidemiology, Biostatistics, and Environmental Health, School of Public Health, University of Memphis, Memphis, TN, United States of America; Northern Arizona University, UNITED STATES OF AMERICA

## Abstract

Mediation analysis is commonly implemented in psychological, epidemiological, and social behavior studies to identify potential factors that mediate associations between exposures and physical or psychological outcomes. Various analytical tools are available to perform mediation analyses, among which Mplus is widely used due to its user-friendly interface. In practice, sumptuous results provided by Mplus, such as the estimated standardized and unstandardized effect sizes, can be difficult for researchers to choose to match their studies. Through a comprehensive review and utilizing findings from a proven study, we proposed guidelines and recommendations to help users select between standardized or unstandardized results based on data attributes and users’ hypotheses. We also provided guidelines to choose from several types of standardized values based on the types of variables, including exposures, mediators, and outcomes.

## Introduction

Mediation analysis is a statistical method where a third hypothetical variable is used to explain the relationship between independent and dependent variables. The basic framework of a mediation analysis consists of a minimum of three variables: the independent variable (*X*), the mediator (*M*), and the dependent variable (*Y*) [1]. The effects of *X* on *Y* include direct, indirect (mediated), and total effects. The total effect is the effect of *X* on *Y*. This total effect can be decomposed into the effect of *X* on *Y*, adjusting the effect of *M*, which is referred to as the direct effect, and the effect of *X* on *Y* through the mediator M which is referred to as the indirect effect.

Different tools and software are available for analyzing and extracting results for mediation analysis. Among them, Mplus is one of the most popular tools, especially for researchers in epidemiology and psychology [2, 3]. Mplus distinguishes itself in the realm of mediation analysis through its robust handling of both observed and latent variables, offering sophisticated methods like structural equation modeling (SEM) and growth curve modeling. It provides detailed output including direct, indirect, and total effects, with advanced options for model estimation and handling missing data [3]. While tools available in SPSS, R, Stata, and SAS offer varying levels of simplicity, user-friendliness, and specific strengths in statistical modeling, Mplus stands out for its comprehensive and intricate analysis capabilities and its richness of different types of results, such as unstandardized results and standardized results.

When using Mplus to conduct mediation analysis, it provides several types of results, such as unstandardized results and standardized results [3]. The debate among researchers in different fields on whether to use standardized or unstandardized effect sizes or regression coefficients [4–6] has been carried on for a long time. Fewer studies have taken the initiative as an effort to provide a resolution to end such a debate.

**Fig 1:**
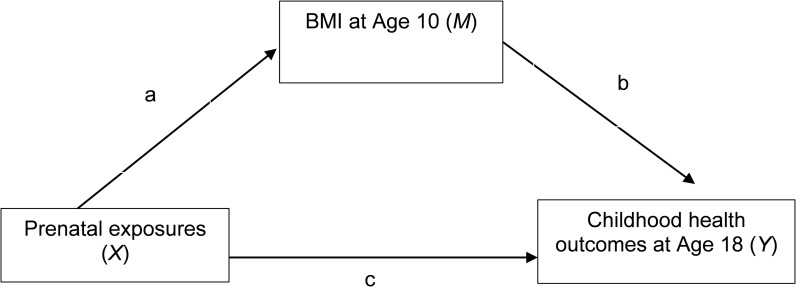
Structure of the mediation analysis assessing the mediation effect of BMI at age 10 on the association of prenatal exposure (maternal BMI) with health outcome at age 18.

To better illustrate different options (standardized/unstandardized) that are available in Mplus, we choose a mediation analysis implemented in a cohort study as an example., which was established on the Isle of Wight (IOWBC) in the United Kingdom [7]. This study aims to assess the mediation effects of BMI (body mass index) at age 10 in the association of prenatal exposure maternal BMI with systolic blood pressure at age 18 (Fig 1). The independent variable (denoted by *X*) is maternal BMI during pregnancy which is continuous, or maternal obesity level, which is binary. The outcome variable (*Y*) is systolic blood pressure at age 18, or whether the subject has high blood pressure defined as systolic blood pressure 130 or higher. BMI at age 10 is included as a potential mediator (*M*), and it is a continuous variable. As shown in Fig 1, parameter *a* denotes the regression coefficient for regressing *M* on *X*, *b* is the regression coefficient when regressing *Y* on *M* adjusting for *X*, and *c* is the regression coefficient when regressing Y on X with M in the model. When Y is continuous, the indirect effect is the product of *a* and *b*, *a* × *b*. When Y is binary, it is formulated into a latent continuous indicator using logit link or binary link [8, 9]. Logistic or probit regression is used to model the outcome probabilities. The indirect effect is calculated by the differences of predicted probabilities or risk ratios [8, 10, 11].

We use data from a birth cohort established on the Isle of Wight (IOWBC) in the United Kingdom [7] to fit the aforementioned mediation analysis model in Mplus. The output given by Mplus includes a set of results, each from a specific standardization method. The results are summarized in Tables 1–3. In addition to unstandardized results (the last column panel in the tables), results from two standardization methods are also included. The first approach is labeled as *STDYX* such that data of both exposure variables (*X*) and the outcome variables (*Y*) are standardized. The second approach is *STDY*, and only the outcome variable (*Y*) data are standardized. In both approaches, *STDYX* and *STDY*, the mediator (*M*) is always standardized.

**Table 1:** Summary of results from Mplus for continuous outcome (systolic blood pressure at age 18).

**Continuous exposure (maternal bmi)**									
	Results under STDYX			Results under STDY			Results without standardization		
	Estimate	S.E.*	P-value	Estimate	S.E.	P-value	Estimate	S.E.*	P-value
***a***	0.349	0.042	0	0.084	0.010	0	0.251	0.032	0
***b***	0.199	0.056	0	0.199	0.056	0	0.794	0.222	0
***c***	0.066	0.042	0.115	0.016	0.010	0.113	0.189	0.119	0.111
**Indirect effect**	0.069	0.021	0.001	0.017	0.005	0.001	0.199	0.062	0.001
**Binary exposure (maternal obesity category)**									
	Results under STDYX			Results under STDY			Results without standardization		
	Estimate	S.E.*	P-value	Estimate	S.E.	P-value	Estimate	S.E.*	P-value
***a***	0.247	0.040	0	0.524	0.086	0	1.556	0.268	0
***b***	0.210	0.055	0	0.210	0.055	0	0.844	0.220	0
***c***	0.021	0.041	0.600	0.045	0.086	0.601	0.539	1.027	0.600
**Indirect effect**	0.052	0.013	0.002	0.110	0.035	0.002	1.313	0.425	0.002

Note:

*S.E. Standard error

** in Mplus, it only prints out three digits after the decimal place and thus all zero values indicate that the number is <0.001.

**Table 2:** Summary of results from Mplus for binary outcome (systolic blood pressure category at age 18) by probit regression analysis with mean and variance-adjusted weighted least squares (WLSMV) estimator.

**For continuous exposure (maternal bmi)**									
	Results under STDYX			Results under STDY			Results without standardization		
	Estimate	S.E.*	P-value	Estimate	S.E.*	P-value	Estimate	S.E.*	P-value
***a***	0.344	0.042	0	0.083	0.010	0	0.247	0.032	0
***b***	0.284	0.073	0	0.284	0.073	0.016	0.096	0.024	0
***c***	0.012	0.067	0.861	0.003	0.016	0.861	0.003	0.016	0.862
**Indirect effect**	0.098	0.028	0.001	0.024	0.007	0	0.024	0.007	0.001
**For Binary exposure (maternal obesity category)**									
	Results under STDYX			Results under STDY			Results without standardization		
	Estimate	S.E.*	P-value	Estimate	S.E.*	P-value	Estimate	S.E.*	P-value
***a***	0.241	0.041	0	0.512	0.086	0	1.517	0.268	0
***b***	0.287	0.072	0	0.287	0.072	0	0.097	0.024	0
***c***	-0.001	0.067	0.992	-0.001	0.143	0.992	-0.001	0.144	0.992
**Indirect effect**	0.069	0.022	0.002	0.147	0.047	0.002	0.147	0.047	0.002

Note:

*S.E. Standard error

** in Mplus, it only prints out three digits after the decimal place and thus all zero values indicate that the number is <0.001.

**Table 3:** Summary of results from Mplus for binary outcome (systolic blood pressure category at age 18) by logit regression analysis with maximum likelihood (ML) estimator.

**For continuous exposure (maternal bmi)**									
	Results under STDYX			Results under STDY			Results without standardization		
	Estimate	S.E.*	P-value	Estimate	S.E.*	P-value	Estimate	S.E.*	P-value
***a***	0.347	0.041	0	0.084	0.010	0	0.249	0.032	0
***b***	0.289	0.073	0	0.289	0.073	0	0.184	0.049	0
***c***	0.006	0.070	0.930	0.001	0.017	0.930	0.003	0.032	0.931
**Indirect effect**	0.100	0.028	0	0.024	0.007	0	0.046	0.014	0.001
**For Binary exposure (maternal obesity category)**									
	Results under STDYX			Results under STDY			Results without standardization		
	Estimate	S.E.*	P-value	Estimate	S.E.*	P-value	Estimate	S.E.*	P-value
***a***	0.246	0.040	0	0.522	0.086	0	1.549	0.267	0
***b***	0.292	0.072	0	0.292	0.072	0	0.187	0.050	0
***c***	-0.003	0.071	0.964	-0.007	0.152	0.964	-0.013	0.291	0.964
**Indirect effect**	0.072	0.023	0.002	0.153	0.048	0.002	0.289	0.098	0.003

Note:

*S.E. Standard error

** in Mplus, it only prints out three digits after the decimal place and thus all zero values indicate that the number is <0.001.

The tables (Tables 1–3) show that regression coefficients vary with different standardization approaches, although the p-values are consistent. For example, the regression estimate (a) in Table 1 for continuous exposure is 0.349 from *STDYX*. In contrast, it is 0.084 from *STDY* which is a 76% decrease. Similarly, the indirect effect is significant in Table 1 for binary exposure but indirect effect estimate from *STDYX* is 0.052 whereas from *STDY* it is 0.110 (112% increase from *STDYX*) and 1.313 (almost 1000% increase from *STDY*) in the original scale. Another example would be, as shown in Table 2 (probit analysis with WLSMV estimator), when the outcome variable is binary and the exposure variable is also binary, the regression coefficient for indirect effect in the original scale is 0.147, but 0.289 (almost 100% increase) in Table 3 (logit analysis with MLR estimator) for the same variables and path. In practice, among these different results, which one should be recommended? Between these two types of coefficients, some researchers strongly discouraged reporting (only) standardized coefficients when the exposure variable is dichotomous [1]. Hayes suggests reporting either unstandardized or both unstandardized and partially standardized coefficients [1].

In this article, we attempt to summarize several criteria to help users to decide whether to choose standardized results or not. The criteria are constructed based on two considerations: the study’s goal and the type of variables (for the outcome, explanatory, mediator, and latent variables) used in mediation analysis. In the following, we first introduce the advantages and disadvantages of standardizing of the data, followed by a proposed guideline for selecting appropriate mediation results.

## Advantages and disadvantages of standardization

### 1) Advantages of standardization

When the primary goal of a study is to find the strongest predictor among multiple candidates for the dependent variables, standardized coefficients (i.e., coefficients estimated from standardized data) generally give the best results. Such coefficients can be used to rank the importance of candidate predictors, e.g., using the absolute value of the estimated regression coefficients, and the variable with the largest absolute value of regression coefficients is deemed as the most important variable. Unstandardized coefficients (i.e., estimates based on unstandardized data) do not own this advantage since they are scale dependent.

In addition, the independence of standardized estimate effects on their original scales makes it possible to interpret the findings without detailed knowledge of those scales [12]. Researchers will also want to determine the statistical and practical significance of indirect effects in practice. This is where standardization comes in handy. Often, it is of great interest to report the proportion of the total effect due to indirect effects or equivalently, to report the ratio between the indirect and the direct effect. The unitless standardized coefficients make the calculations meaningful. These proportions or ratios are often used as effect sizes to assess the contribution of indirect effects [13, 14]. It is worth pointing out that, in this case, the same direction between direct and indirect effect coefficients is required. If the indirect and direct effects have different signs, then the mediation effect is called inconsistent mediation [15]. Thus, the use of proportions for indirect effect size calculations may give misleading estimates of an effect’s magnitude and importance [16].

### 2) Disadvantages of standardization

Suppose the exposure variables in a model are based on measurement scales such as a Likert scale or ratio scale. In that case, coefficients estimated based on unstandardized data have consistent units with those of the exposure and dependent variables. Such consistency will ensure meaningful interpretations of the estimated coefficients. On the other hand, standardizing this type of variable results in a different scale and makes it hard to interpret with respect to the original scales [4]. Thus, using regression coefficients from standardized data may lead to a biased understanding of the variable’s effect size compared with other variables.

It’s important to note that standardized coefficients can sometimes give a misleading of the importance of certain variables, as they transform the coefficients to a common scale, obscuring the true nature of relationships in the data [17]. This potential for misleading estimates is further compounded by the fact that standardized coefficients can sometimes lead to an exaggerated sense of importance for variables with larger scales, due to their normalization to a common scale. In addition, reliance on standardized coefficients can mask issues of multicollinearity, where predictor variables are correlated with each other. This correlation can inflate coefficients and give a false impression of their importance [18].

## When to do standardization? / Suggested guidelines

Based on the discussion above, we propose the following recommendations to help researchers to decide whether or not to choose standardized regression coefficients when conducting mediation analyses. These recommendations are driven by the goal of the studies, or the types of variables used.

### 1) The goal of the study

If the study is to select the strongest predictor rather than interpreting the change of outcome variable due to change of predictors, standardized coefficients give the best results. Suppose the study aims to calculate indirect effect through point estimates of proportion mediated or ratio mediated for a sample size smaller than 500. In that case, standardized coefficients are shown to be the better choice. On the other hand, if the study relay on more to understand the association of change of outcome variable due to change of predictors and mediators, unstandardized coefficients are more suitable for practically meaningful interpretations.

### 2) The types of variables used

#### a) Binary outcome (*Y*)

For binary outcome variables, standardizing the variables will alter the scales, and thus interpretation of the coefficients can be misleading. Thus, using unstandardized values for regression coefficients will be a better solution. For our study example, from Table 3, we can now use the estimate of the unstandardized regression coefficient of b (-9.085) which is very different in magnitude compared to the standardized regression coefficient (-.208).

Both probit and logit regression models are used when the outcome variable is dichotomous. As a related topic, here we propose a suggestion on the selection between probit and logit regressions. In the logit model, the cumulative distribution function of the logistic distribution is used, and in the probit model, the cumulative distribution function of the standard normal distribution is used.

It has been demonstrated that maximum likelihood with a robust standard error estimator (MLR estimator used in the logit model) performs similarly to the weighted least square mean and variance adjusted estimator (WLSMV estimator used in the probit model) [19]. It is increasingly agreed that WLSMV works better in the case of categorical variables with a sample size of more than 200. But for smaller sample sizes, compared with WLSMV, MLR has lower statistical power but better control of Type I error [19]. The Mplus uses the WLSMV estimator (probit model) as a default when dealing with categorical outcomes.

As demonstrated in our study example, Tables 2 and 3 display results from probit and logit analysis, respectively. Although most results between these two models are similar, some are different in parameter estimates and p-values for *STDYX* and *STDY*. Based on the discussion above, for this example, results from unstandardized data inferred based on probit regression (WLSMV estimator) are most appropriate and are suggested to use.

#### b) Continuous outcome (*Y*) and categorical exposure (*X*)

When *X* is binary, we can use *STDY* for regression of *Y* on *X* or *M* on *X*. *STDYX* is not recommended since *X* is binary. When *STDY* is used to estimate parameters and *Y* is regressed on *M*, both *Y* and *M* data are standardized using *STDY* since *M* is a dependent variable in the regression of *M* on *X* [20]. If the researchers prefer to report standardized results, results from *STDY* are more appropriate and easier to interpret when covariates are binary.

#### c) Continuous outcome (*Y*) and continuous exposure (*X*)

Under this setting, both results from unstandardized and standardized data are applicable. Standardized results are recommended when the contribution of indirect effects toward total effects is an essential component of data analyses. In the situation of standardizing the data, we have multiple choices. We can use *STDYX* to standardize data when regressing *M* on *X* (coefficient a in Fig 1). *STDYX* can also be applied when regressing *Y* on *M* (coefficient b in Fig 1) if the outcome is also continuous. Overall, the regression coefficient and p-value of the indirect effect given by *STDYX* are appropriate to be reported for decision-making. *STDY* is not suggested in this case, given that both *X* and *Y* are continuous. Just standardizing the dependent variable can cause misleading results. As shown in our Mplus results in Table 2, we can now use the STDYX regression coefficient estimate for a (-0.02) compared to the STDY estimate (0) clearing out the confusion between the direction of estimates. Because of the implementation of *STDYX*, users are suggested to be cautious when interpreting the coefficients since both scales of *Y* and *X* have been changed.

In studies with exposure variables in the Likert scale or ratio scale, unstandardized coefficients are also advised if dichotomous covariates are included in mediation analysis.

**Fig 2:**
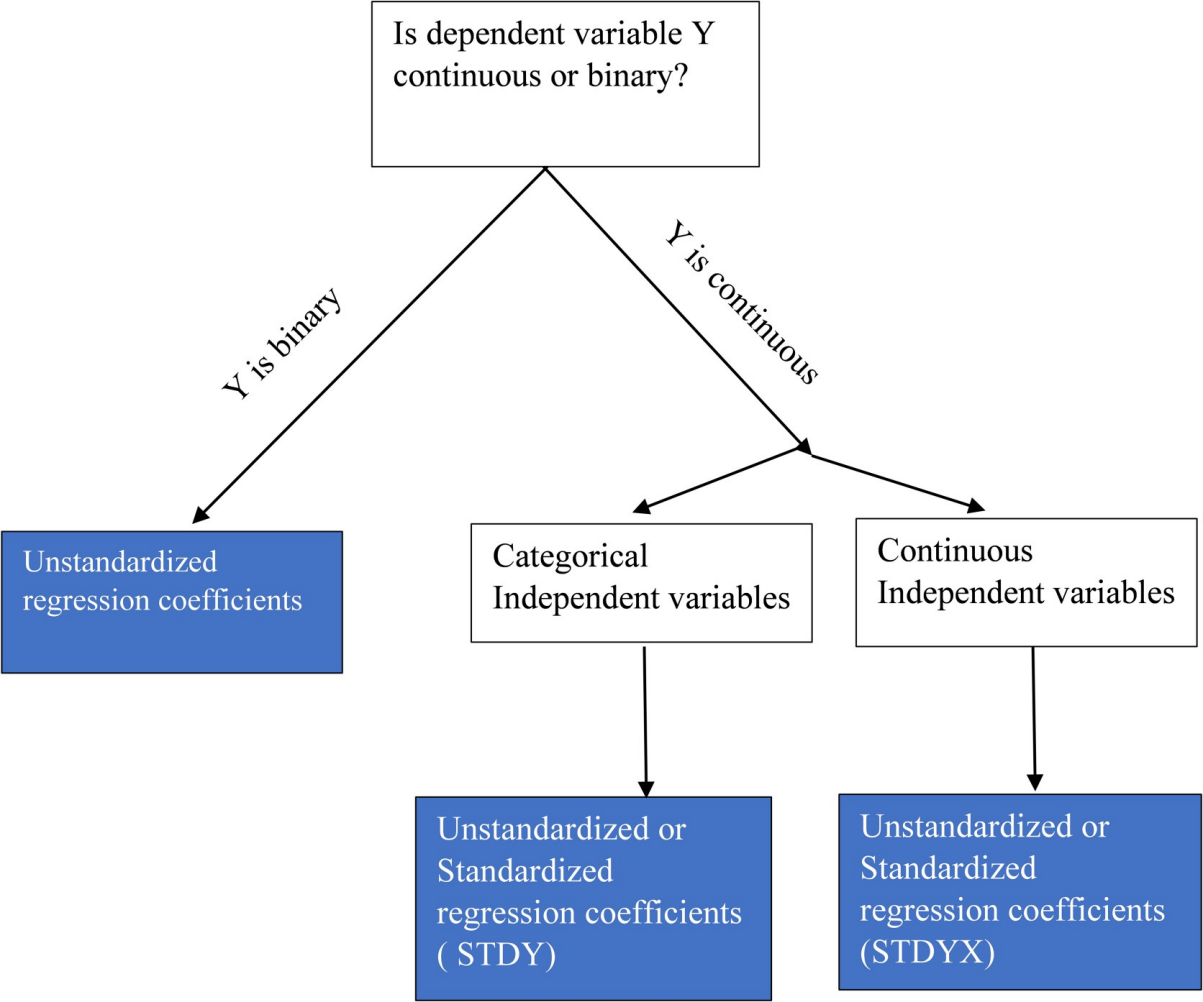
Flowchart of selection of standardized or unstandardized regression coefficients based on the types of variables used in the mediation analysis.

Overall, based on the types of variables, we have developed a straightforward flowchart to assist researchers on the selection of standardized or unstandardized regression coefficients, aligning with our guidelines (Fig 2).

## Discussion

We summarized the pros and cons when using standardized or unstandardized regression coefficients in mediation analysis. We then suggested general guidelines for specific circumstances based on the study’s goal and the types of variables used in the study, accompanied by a flow chat to further assist researchers’ selection. It worth noted that in all the cases discussed in this article, mediating variables are continuous. Binary mediators are not in the scope of this paper.

When we construct confidence intervals for indirect effects using unstandardized coefficients, the distribution of ab (the product of regression coefficients a and b) is assumed to be normal. Although in large samples, this is a safe assumption to make, in real-life data analysis situations, this compromises the validity of the inference as ab tends to be positively skewed and highly leptokurtic [6]. Thus, constructing confidence intervals based on normality can generate misleading inferences. One way to recover from this is to use bootstrap confidence intervals constructed via unstandardized coefficients, which use ‘the empirical distribution of the statistics to approximate the underlying distribution of the statistics’ [12, 21]. This procedure produces more reliable confidence intervals. It is worth noting that the statistical testing power tends to be lower than optimal when standardization is not used [21, 22].

In the current study, both continuous and binary exposure or outcome variable are discussed. In certain research contexts, converting continuous physical or biological measures, such as BMI or blood pressure, into binary variables can be driven by substantive reasons. Clinically, binary classifications align with thresholds used to define health conditions, such as obesity (BMI ≥ 30), which can guide targeted interventions and inform public health strategies. For example, in our case study, when studying the impact of maternal BMI on child health outcomes, using a binary classification (obese vs. non-obese) may be more relevant for identifying at-risk populations and implementing appropriate interventions. On the other hand, treating BMI as a continuous variable is beneficial for capturing the extent of physiological changes and providing detailed insights into how BMI fluctuates with exposures or interventions. Additionally, in cases where BMI is suspected to have measurement errors, converting it to a binary variable and treating it as a single-indicator latent variable via logit or probit links can offer more robust estimates and better capture underlying clinical risks [9]. Thus, the choice between continuous and binary measures should be driven by the specific research question and the practical context, whether it involves understanding specific physiological changes or assessing clinical risks. Our practical data analyses and guidelines show that, whether we use binary or continuous exposure, when the outcome is binary, using unstandardized coefficient is the best approach. But, when the outcome is continuous and we use a converted binary physiological measure (such as maternal obesity category), we can provide STDY regression estimate as more appropriate and interpretation worthy.

When dealing with binary outcome variables in Mplus, the standardization of effect estimates is generally not necessary. This is because the effects are already on a probability scale, inherently standardizing the coefficients with respect to the dependent variable (MUTHEN 2015). We emphasize that when the outcome variable is binary, researchers should rely on unstandardized data to obtain meaningful regression coefficients. Specifically, we recommend using unstandardized estimates from probit regression (WLSMV estimator) rather than logit regression (ML estimator) due to the proven success of the WLSMV estimator in handling sample sizes larger than 200. This approach ensures more accurate and reliable parameter estimates, leveraging the strengths of the WLSMV estimator in managing categorical outcomes and providing robust results.

Additionally, this study exclusively focuses on continuous mediators. Mediation analysis involving binary mediators are more complexed and not fully developed, especially when the outcome is also binary [23]. The estimation of indirect and total effect can be inconsistent when using different approaches [24]. To maintain clarity and focus, we chose to limit our discussion to continuous mediators.

Lastly, we would like to point out that standardization is also used in mediation analysis with latent variables [8, 25]. Mplus uses the option ‘std’ to show standardized values for latent variables. When latent variables are used in the model, they need to be scaled to estimate the model’s parameters [22]. However, scaling latent variables while simultaneously scaling observed variables can cause estimated regression coefficients to depend on the metric of the observed variables and the scaling method of the latent variables. Using standardized regression coefficients can outmaneuver the problem of arbitrary metrics [26], and they are invariant towards scaling methods for latent variables [27].

Furthermore, the concepts and guidelines presented in the current paper, which involve the use of standardized or unstandardized regression coefficients in mediation analysis, are illustrated using Mplus. It’s important to note that these guidelines are not limited to Mplus. The general principles are applicable to mediation analyses that are implemented in other software platforms.
